# High gain chopper supplied from PV system to fed synchronous reluctance motor drive for pumping water application

**DOI:** 10.1038/s41598-022-19671-x

**Published:** 2022-09-15

**Authors:** Z. M. Salem Elbarbary, Khalid Mehmood Cheema, Saad F. Al-Gahtani, Ragab A. El-Sehiemy

**Affiliations:** 1 Electrical Engineering Department, College of Engineering, King Khalid University, Abha, 61421 Saudi Arabia; 2 Electric Engineering Department, Faculty of Engineering, Kafrelsheikh University, Kafrelsheikh, 33516 Egypt; 3 School of Electrical Engineering, Southeast University, Nanjing, 210096 China; 4 Department of Electronic Engineering, Fatima Jinnah Women University, Rawalpind, 46000 Pakistan

**Keywords:** Fluid dynamics, Computational science, Information theory and computation

## Abstract

Power generation for renewable energy sources increased rapidly during last few years. Similarly, the high gain dc–dc boost choppers are taking place of conventional power converters used for photovoltaic (PV) appliances. Researchers are developing different methods in order to provide high voltage gain, low ripple, reduced switch stress, low converter costs, and minimized variations of PV operating points. This study proposes a two-stage converter for a freestanding water pumping motor drive power by solar PV system. According to the proposed system, at first, a high gain (HG) cell and a DC-to-DC boost converter are combined to increase the PV voltage to high levels. Later on, the resulting dc voltage feds a three-phase synchronous reluctance motor drive that operates centrifugal pump load. The perturb and observe approach is utilized to get the maximum power out of the solar PV module. Moreover, indirect field-oriented control is implemented to accomplish smooth starting of synchronous reluctance motor. In order to validate the effectiveness of proposed technique, a MATLAB/Simulink environment-based simulation setup along with an experimental prototype is developed. Additionally, various cases are considered based on different operating conditions and irradiance levels to collect and analyse the results.

## Introduction

Undoubtedly, the development of renewable energy resources helps power system operators and planners increase their applications in the industry. Solar power water pumping systems have become popular and attractive in remote areas, particularly where there is no access to a conventional power grid^1^. Though, the solar power water pumping system has some limitations, such as it cannot pump water at night or on cloudy days. However, these limitations can be overcome by installing the energy storage system with the PV generation units^2^. But the batteries in energy storage systems have their own drawbacks, such as shorter life and uneconomical. Additionally, batteries require continuous maintenance and service, increasing overall expenditure^3^. To overcome these disadvantages, it is necessary to pump the water during the daytime and store the excessive water in special reservoirs. The stored water can be utilized at night or on cloudy days for irrigation or other necessary utilities^4^, The solar photovoltaic array functions as a major energy source; in contrast, a battery is employed as a backup supply and is charged by the SPV array when the pump is not running or is running at a reduced rating^5–7^.

As the integration and implementation of solar power water pumping systems are increasing, the researchers are focusing on improving the overall reliability and effectiveness of these systems and developing economic and simple control approaches for the drive unit. It is apparent from various sources that the drive unit utilized for water pumping accounts for roughly 1/3 of the overall system expenditures^8^. The performance of the drive unit has a direct impact on system effectiveness and efficiency. Therefore, an appropriate and efficient drive unit for a solar power water pumping system is critical^9^.

Generally, solar power water pumping systems utilize induction motors (IM), conventional DC motors, switched reluctance motors, and Brushless DC motors (BLDC). Each type of motor has its advantages and disadvantages; for example, IM is robust and cost-effective, but it has drawbacks in application solar power water pumping, especially for partial wattage systems^10^. Subsequently, conventional DC motors have low efficiency, and they require mechanical shifters along with carbon brushes for operation, which requires regular maintenance^11^. The frequent maintenance and excitation losses cause process interruption and low efficiency^12^. However, switched reluctance motor has the most basic ruggedness, and it overcomes these troubles. In Refs.^13–16^, researchers discuss the benefits of synchronous reluctance motors with adjustable-speed drives. The researchers concluded that the synchronous reluctance motors required a simple rotor structure, minimum inertia, and an effortless speed control unit without sensors. Additionally, synchronous reluctance motors do not need a rotor cage in speed-controlled drives, and their resistive losses are minimum. Furthermore, compared to the synchronous permanent-magnet motors, synchronous reluctance motors feature a simple field weakening process, and it does not require costly magnets.

Consequently, the addition of a DC–DC converter with the inverter reduces the flexibility of PV power generation^17,18^. Because there are more switching devices and passive elements in this two-stage power conversion, the voltage profile might be stopped immediately^19^. Therefore, traditional boost converters require a large duty cycle to achieve high-level voltage.

The creation of a high-performance, high step-up dc–dc converter is a typical necessity for the applications mentioned above. For instance, an inverter must be required to raise the 12-V vehicle battery to roughly 100 V in order to operate a small-wattage metal halide light HID (commonly referred to as a xenon lamp) with a 35 W rated power for automobile headlamps. Another potential usage is to transform the fuel cell's low-dc energy source (25–45 V) into an appropriate utilisation current, voltage, and frequency for utility loads. Thus, in order to be reversed into ac power for the grid, this low voltage range must be converted into a sufficient dc link voltage (350–400 VDC).But high gain places a lot of stress on the dv/dt switch. Moreover, the redundant resistances are used to enhance the high-voltage gain performance, and reverse recovery diode is incorporated to reduce huge current ripples^20,21^. In order to achieve a sustainable and effective high input voltage, researchers proposed various DC-DC converters in the literature^21–23^. However, these DC-DC converters require extra components, such as special types of inductors and capacitors, to gain a specific ratio of voltage^24–26^.

Considering the literature, in Refs.^27,28^ researchers proposed a high gain boost convertor by using a conventional induction motor. Consequently, in Ref.^29^ the authors developed a technique by using the capacitor-based switching method to achieve high gain from boost converters. Similarly, in Ref.^30^ authors presented the PWM technique for high gain boost resonant converter. However, in this paper, the synchronous reluctance motors are used for the effective driving system, and the key contributions of this paper are as follows:

1. Determine the maximum power point of PV at various levels of irradiation and temperature.
2. The high gain (HG) cell and a DC-to-DC boost converter are combined with increasing the PV voltage to high levels, to separate the system from the array when there is low light. The water pumping system is then powered by the backup battery. The next section provides a thorough explanation of the proposed system.
3. The resulting dc voltage feds a three-phase synchronous reluctance motor drive that operates centrifugal pump load.
4. The perturb and observe approach is utilized to get the maximum power out of the solar PV module, and indirect field-oriented control is implemented to accomplish smooth starting of synchronous reluctance motor
5. The experimental analysis is performed that confirms the effectiveness of the proposed system.

The rest parts of this paper are organized as follows: In “Photovoltaic high gain converter fed three-phase SynRM drive system”, a detailed description of the proposed driving system is presented. “Dynamic model of 3-phase synchronous reluctance motor” describes the Dynamic Model of 3-phase synchronous reluctance Motor. “Experimental setup of the tested system” shows the experimental implementation of the proposed scheme. Simulation and experiential results are recorded in “Results and discussions”, based on the obtained results.

## Photovoltaic high gain converter fed three-phase SynRM drive system

**Figure 1:**
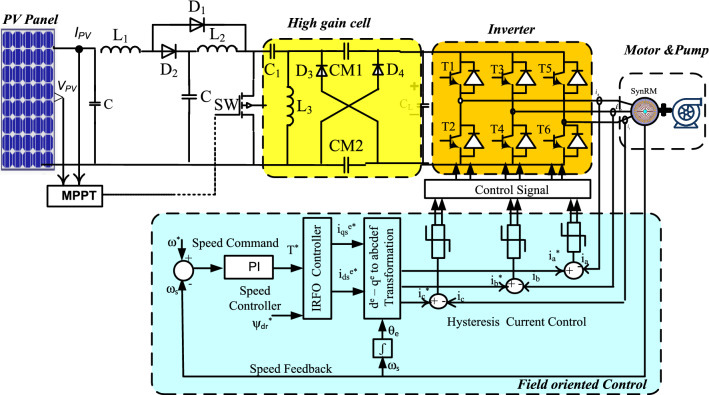
Circuit configuration of the PV high gain converter fed three-phase SYNRM drive system.

The anticipated DC–DC transformer-less high step-up converter topology for photovoltaic (PV) supply three-phase SynRM drive pumping load is illustrated in Fig. 1. The proposed scheme comprises a PV with a maximum power point tracking system, a DC–DC boost chopper, along a high gain cell. Additionally, a driving scheme is developed by using indirect field-oriented control. The three-phase synchronous reluctance motor drive pumping load is supplied from the converter's output voltage.

A brief description and mathematical analysis of each unit are given below.

### PV Model

**Figure 2:**
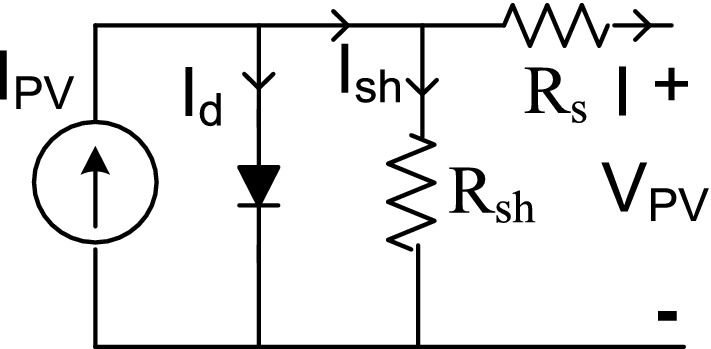
Single diode PV equivalent circuit.

The solar cell is a device that converts photon energy into electricity. In literature, numerous researchers presented and explained various models of solar cells^31–33^. However, a simple and basic single diode model is discussed in this paper, as shown in Fig. 2^34^. The single diode model is relatively simple and highly effective for dynamic modeling of the photovoltaic system. Considering the single diode model of Fig. 2, the load current (I) is obtained from Eq. (1) as:

1 $${\text{I }} = {\text{ I}}_{{{\text{PV}}}} - {\text{ I}}_{{\text{d}}} ,$$

where I_d_ and I_PV_ are diode current and photovoltaic current, respectively.

The current source (I_PV_) declares the cell photovoltaic current presented in Eq. (2) as:

2 $${\text{I}}_{{{\text{PV}}}} = \, \left( {{\text{I}}_{{{\text{SC}}}} + \, \beta \, \left( {{\text{T}} - {298}} \right)} \right)\delta /{ 2}000,$$

where, I_sc_ is the short circuit current, *β* is the short circuit current of a cell at 250 °C and 1000 W/m^2^, T is the operating temperature in kelvin, and δ is the solar irradiance. The shunt (R_sh_) and series (R_s_) resistance, as shown in the simple equivalent circuit of the solar cell in Fig. 2 are identified as the inherent resistance of the cell. The value of the shunt resistance is significant, while series resistance is small and can be ignored. Therefore, the output current for the solar cell is given as:

3 $$I={I}_{PV}-{(I}_{d}+{I}_{sh}).$$

The shunt current computed as:

4 $${I}_{sh}=\frac{{V}_{pv}-I{R}_{s}}{{R}_{sh}}.$$

The output voltage of the cell is given as in the following equation:

5 $${V}_{pv}={V}_{d}-I{R}_{s}.$$

### MPPT detection scheme

Several environmental constraints, such as subjective shading, dust, and wind problems, affect PV power present occasional performance. Based on the site and period, Sunshine intensity contrasts significantly; this creates deviation in the temperature of the cell and solar radiation. Total resistance and temperature impact the construct of the inverter beside the system. In order to obtain the highest power from solar modules at any time, solar converters are utilized to the solar panel to its highest voltage and provide all the efficient power. The MPPT dupes the solar panels into obtaining a variable voltage and current allowing further power to the load. The MPPT examines the output current and voltage from the solar panel and selects the operation point to deliver the maximum power offered to the load. To improve PV efficiency, the MPPT must precisely follow the constantly adjusting operating point where the power is at its maximum. Numerous methods have been created for finding the maximum power point of PV These systems vary in complication, speed, convergence, efficiency cost, and sensors required. Traditional P&O has significant benefits, and many studies adapted it; the fluctuation challenge and MPP tracing in the rapid climate change are tricky challenges.

#### Perturb observe scheme

**Figure 3:**
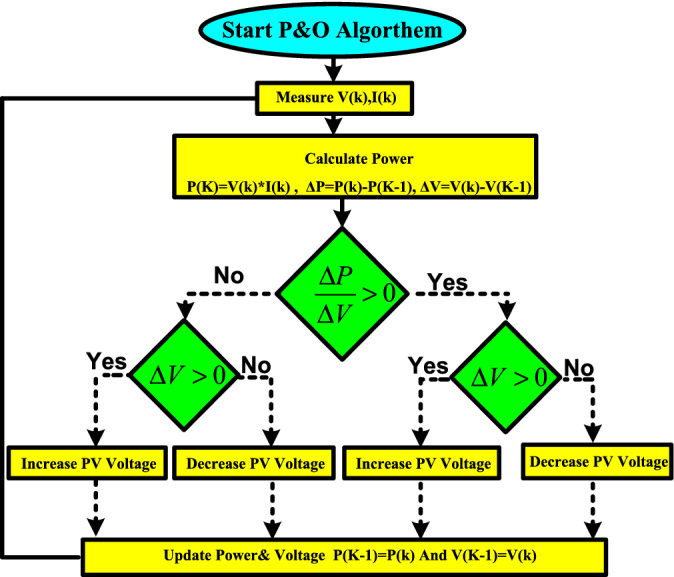
Flowchart of perturb and observe scheme.

**Figure 4:**
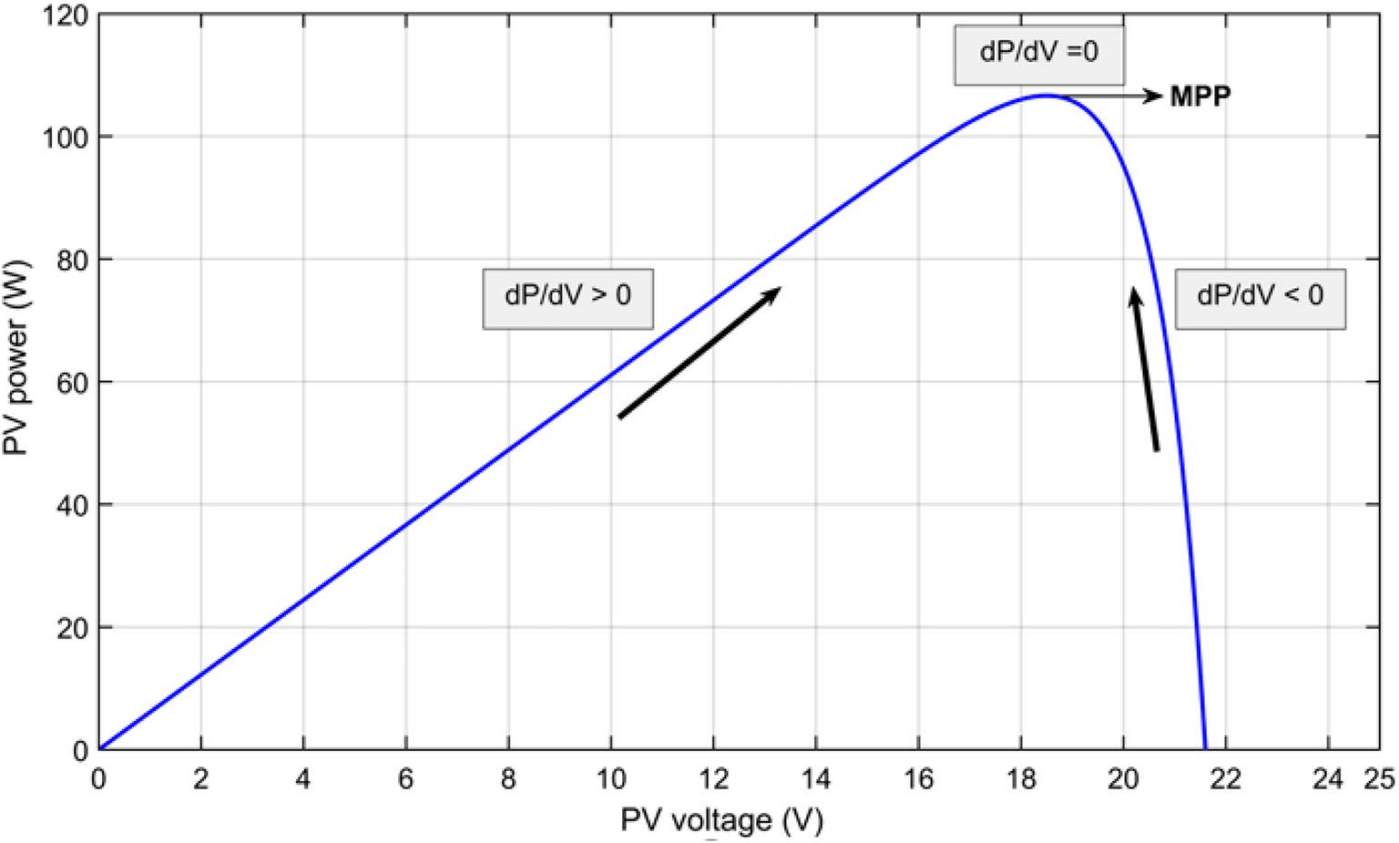
Power against voltage for P&O Algorithm**.**

P&O system is the easiest, gratuitous, widespread, and practically valid in preparation with efficiency up to 96.5%^3^. But it is not tough to trace the correct MPP at quick fluctuations of climate^9,16,21,35^. The procedure takes its data from the real operation point of the solar PV array (i.e., voltage, V_pv_ and current, I_PV_) to examine the P–V curve to acquire MPP, as shown in Fig. 1. The scanning of the P–V curve is done by modifying the operational point (V_PV_ or I_PV_), which is recognized as perturbation step, and subsequently measuring the variation in PV power (∆P), which is well-known as the observation step. The flowchart of the common P&O system is shown in Fig. 3. The consequential change of PV power is stated as:^1^,

- If: $${(}{\raise0.7ex\hbox{${{\Delta P}}$} \!\mathord{\left/ {\vphantom {{{\Delta P}} {{\Delta V}}}}\right.\kern-\nulldelimiterspace} \!\lower0.7ex\hbox{${{\Delta V}}$}}{ > 0)}$$, the perturbation of voltage must be increased in the MPP direction.
- If: $${(}{\raise0.7ex\hbox{${{\Delta P}}$} \!\mathord{\left/ {\vphantom {{{\Delta P}} {{\Delta V}}}}\right.\kern-\nulldelimiterspace} \!\lower0.7ex\hbox{${{\Delta V}}$}}{ < 0)}$$, the perturbation of voltage must be decreased in the MPP direction. The procedure is repetitive till is attained to MPP wherever $${\raise0.7ex\hbox{${{\Delta P}}$} \!\mathord{\left/ {\vphantom {{{\Delta P}} {{\Delta V}}}}\right.\kern-\nulldelimiterspace} \!\lower0.7ex\hbox{${{\Delta V}}$}}$$ is strong to zero as shown in Fig. 4 this is assured state is labeled steady state. The tracking performance for P&O MPPT technique is assessed using the tracking efficiency which is is described as

$\eta_{{{\text{MPPT}}}} { = }\frac{{\int_{{{\text{t1}}}}^{{{\text{t2}}}} {Pdt} }}{{\int_{{{\text{t1}}}}^{{{\text{t2}}}} {P_{Max} dt} }}$ where P is the array output power and Pmax is the theoretical maximum array power, and t1 and t2 are the system's start-up and shut-down times, respectively. The tracking efficiency for P&O arounf 96%^36^.

The P&O maintains perturbing the scheme to identify a difference in the MPP (affected by a variation in the environmental situations or load), which activates a new check. Generally, this procedure produces the PV approach's operational point to fluctuate throughout MPP.

### DC–DC high gain converter

The conservative PWM step-up dc–dc chopper schemes functional in the continuous conduction approach, which afford a high dc voltage gain, are restricted in applied tenders by the fastener-up state and the degradation in the total converter efficacy as the duty ratio methods. Table 1 describes the general categories and key traits of high gain step-up DC/DC chopper Converter constructions and their properties^37–39^.

**Table 1:** DC/DC converter constructions and their features.

DC/DC chopper class	Features
Non-isolated	Frequently easy constructs Suitable for applications of low to medium power The input and output are electrically connected
Isolated	Decreased noise and EMI difficulties Acceptable for high power concentrations Simple execution of several output topologies with (+) and/or (−) voltages Go through the majority function grid requirements Require accurate paired magnetic strategy for high voltage gain
Unidirectional	There is only one direction for power flow Simple control and modulation When compared to bidirectional, it's less complicated and expensive
Bidirectional	Suitable for regenerative applications, with both forward and reverse power flow Facility FET driver and control units are required
Voltage-supplied	High input current ripple Buck traits that are present at birth. A quick and dynamic response
Current supplied	Input current that is constant and has a slight ripple Boosting properties that are built in The dynamic is slow due to the input inductor
Hard switched	Switching loss is high—power density is low High EMI at switching transitions due to high dv/dt and di/dt Switching frequency is limited. Frequently inefficient
Soft-switched	Close to zero switching loss (ZVS and ZCS.) Partially complicated analysis High-level of switching frequency Enhanced power density with high efficiency
Non-smallest phase	Decrease the dynamic response time Control design is frequently difficult
Smallest phase	Easy control design, with a fast dynamic response and a significant stability

**Figure 5:**
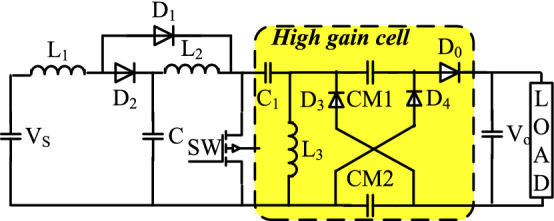
Proposed non-isolated high step-up DC/DC converter.

Figure 5 illustrates the topology of the proposed transformer-less high gain DC/DC converter circuit. The main source is a low DC input voltage [V_S_], and only a full controlled single switch (SW), [IGBT or MOSFET] together with three diodes [D_1_ and D_2_] are utilized. With the output diode [D_0_] and capacitor [C_0_], three inductors [L_1_, L_2_, and L_3_], and five capacitors [C, C_1_, CCM_1_, CCM_2_, and C_0_] are also used. A capacitor-diode-based quadratic boost converter with blending and integration is presented to increase the static voltage gain. Its parts reduce switch stress. The DC/DC converter operates more rapidly with this design than with other converter topologies. L_1_ inductor inrush current is additionally reduced.

It also reduces switching stress^35,37^. The proposed high-gain dc–dc converter can offer voltage gains of 10–30 times the input voltage at the least possible duty ratio. In order to simplify the converter analysis, it is assumed that ideal semiconductor switches that result in 100% efficiency of the chopper with constant switching frequency. Each capacitor is designed to have a minimum voltage ripple^40^.

### Converter modes of operation

**Figure 6:**
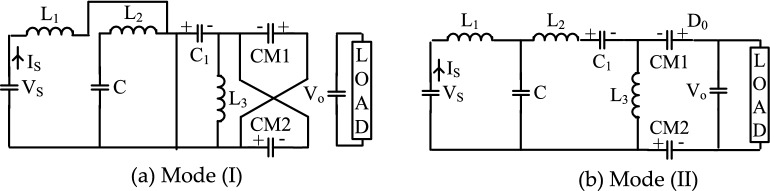
Proposed converter mode of operations.

The suggested topology has two operating modes, Fig. 6a,b show two modes of operation:

- Mode (I) at the power switch [SW-ON]
- Mode (II) at the power switch [SW-OFF].

These modes are identified as follows:

The current ripple across the inductor (L1) is neglected in CCM. As a result, the circuit works as follows. When the switch (SW) is turned on, negative voltage of (VC) across diodes D1 and D2 causes them to switch off. As a result, the series capacitors (C) are charging the output capacitor(C0) and the load at this point. The switch (SW) is switched off at the end of this mode, and both diodes D1 and D2 are instantaneously turned on, giving a pass for inductor current (IL), and (D3) is turned off by the negative voltage (VCM-V0). During this mode, each capacitor (C) is charged to half its input current value (IS) before being released to provide the load current.

### Converter analysis

Diode D1, D2, D3, and D4, are forward biased during the period at [SW-ON]. As shown in Fig. 6a, D0 is reverse biased.

**Table:** 

Inductor	During (SW-On)	During (SW-OFF)	
For inductance (L1)	${\mathrm{V}}_{\mathrm{S}}={\mathrm{V}}_{\mathrm{L}1}$	${\mathrm{V}}_{\mathrm{L}1}={\mathrm{V}}_{\mathrm{S}}-{\mathrm{V}}_{\mathrm{C}}$	(6)
For inductance(L2)	${\mathrm{V}}_{\mathrm{C}}={\mathrm{V}}_{\mathrm{L}2}$	${\mathrm{V}}_{\mathrm{L}2}={\mathrm{V}}_{\mathrm{C}}-{\mathrm{V}}_{\mathrm{CM}}$	(7)
For inductance(L3)	${\mathrm{V}}_{\mathrm{CM}}={\mathrm{V}}_{\mathrm{L}3}$	${\mathrm{V}}_{\mathrm{L}3}={2\mathrm{V}}_{\mathrm{CM}}-{\mathrm{V}}_{0}$	(8)

Applying voltage balance

9 $${V}_{C}=\frac{{V}_{S}}{[1-K]} , {V}_{CM1}={V}_{CM2}= \frac{{V}_{S}}{{[1-K]}^{2}}$$

The duty cycle of chopper with high gain cell:

10 $$\frac{{V}_{0}}{{V}_{S}}= \frac{[1-K]}{{[1-K]}^{2}}$$

The suggested converter's duty cycle (D) is a fixed value produced by the PWM generator.

The converter efficiency is (η) = Pout/ Pin.

The formula is () = Po Pin Pin = Po + Ploss (23).

*Iin Vin* = *Io Vo* + *Ploss* (24).

Power losses (Ploss) are equal to all elemental losses (Pin Po).

where every component, such as the switch's frequency switching loses.

Po = IoVo and Po Vo are the output powers.

As a result, the output voltage and power are directly related. As a result, the efficiency alters as the output voltage varies.

### Design of the converter parameters

The following equations are used to construct the proposed non-isolated DC/DC converter that operates in CCM. In order to obtain the best inductor size and losses in the converter, a limiting current ripple (I_L_) (5–10%) of the nominal current is considered in parameter design. As a result, Table 2 shows the design equations for different elements.

**Table 2:** Design of the converter parameters ns.

No	Element	Design equation-unit	No	Element	Design equation
1	Inductor	${L}_{1} =\frac{k.{V}_{S}}{{f}_{s} {\Delta I}_{L1}}$	3	Inductor	${L}_{3}=\frac{D.{Vcm}}{{f}_{s} {\Delta I}_{L3}}$
2	Inductor	${L}_{2}=\frac{D}{{f}_{s} {\Delta I}_{L2}} \left({Vc}-{Vcm}\right)$	4	Capacitor	$C=\frac{{I}_{L} k}{\Delta Vc {f}_{s}}$

where, ${f}_{s}$ is the switching frequency, ${\Delta I}_{L}$ is the ripple current of inductors.

## Dynamic model of 3-phase synchronous reluctance Motor

The 3-phase synchronous reluctance motor dynamic model is described in a synchronous reference frame. The stator and rotor voltages equations are:

11 $${{V}}_{{{{qs}}}} { = R}_{{{s}}} {{i}}_{{{{qs}}}} { + }\frac{{{{d}}\lambda_{{{{qs}}}} }}{{{{dt}}}}{ + \omega }_{{{s}}} \lambda_{{{{ds}}}},$$

12 $${{V}}_{{{{ds}}}} { = R}_{{{s}}} {{i}}_{{{{ds}}}} { + }\frac{{{{d}}\lambda_{{{{ds}}}} }}{{{{dt}}}}{ - \omega }_{{{s}}} \lambda_{{{{qs}}}},$$

13 $${\text{0 = R}}_{{\text{r}}} {\text{i}}_{{{\text{qr}}}} { + }\frac{{{\text{d}}\lambda_{{{\text{qr}}}} }}{{{\text{dt}}}},$$

14 $${\text{0 = R}}_{{\text{r}}} {\text{i}}_{{{\text{dr}}}} { + }\frac{{{\text{d}}\lambda_{{{\text{dr}}}} }}{{{\text{dt}}}},$$

where, $$\lambda_{{{{qs}}}} { = L}_{{{m}}} {(i}_{{{qs }}} { + i}_{{{{qr}}}} ){ + L}_{{{{ls}}}} {{i}}_{{{qs }}}$$ and $$\lambda_{{{{ds}}}} { = L}_{{{m}}} {(i}_{{{ds }}} { + i}_{{{{dr}}}} ){ + L}_{{{{ls}}}} {{i}}_{{{ds }}}.$$.

The mechanical equation of the drive:

15 $${T}_{e}-{T}_{L}=J\frac{d{\omega }_{s}}{dt}+B{\omega }_{s}.$$

The developed electromagnetic torque of motor:

16 $${T}_{d}=\frac{3}{2}\frac{p}{2}\frac{{L}_{m}}{{L}_{r}}\left({I}_{qs}^{e}{\lambda }_{dr}^{e}-{I}_{ds}^{e}{\lambda }_{qr}^{e}\right).$$

The water pumps have the benefit of a non-linear organization between the load torque (*TL*) and square of motor speed^14^ Thus,

17 $${\text{T}}_{{\text{L}}} {\text{ = K}}_{{\text{p}}} {\upomega }_{{\text{s}}}^{2}.$$

**Figure 7:**
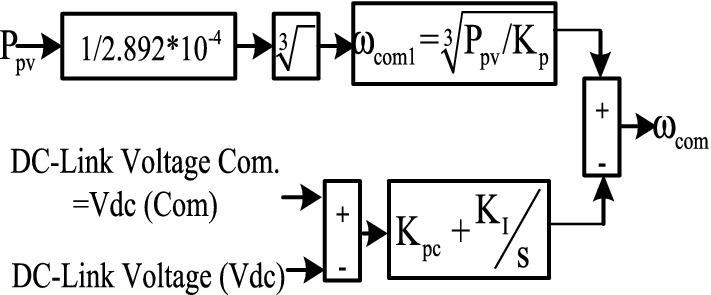
Speed command generation block diagram.

The block diagram of speed command generation as a function of motor speed variations with the changes of PV output power is shown in Fig. 7, and the equation is derived as follow:

18 $$\begin{gathered} K_{p} = \frac{Motor\,rated\,power(W)}{{\omega_{s}^{3} }} \hfill \\ K_{p} = \frac{746 \times 1.5}{{157^{3} }} = 2.892 \times 10^{ - 4} \hfill \\ \omega_{1} = \sqrt[3]{{\frac{{P_{pv} }}{{K_{p} }}}}, \hfill \\ \end{gathered}$$

19 $$\begin{gathered} {\text{V}}_{{{\text{dce}}}} {\text{ = V}}_{{\text{dc }}} {\text{(com) - V}}_{{{\text{dc}}}} \hfill \\ \omega_{{{\text{com2}}}} {\text{ = (K}}_{{\text{pc }}} { + }\frac{{{\text{K}}_{{\text{I}}} }}{{\text{s}}}{)} \times {\text{V}}_{{{\text{dce}}}} \, \hfill \\ \omega_{{{\text{com}}}}\,\, { = }\,\,\omega_{{{\text{com1}}}} + \omega_{{{\text{com2}}}}. \hfill \\ \end{gathered}$$

## Experimental Setup of the tested system

**Figure 8:**
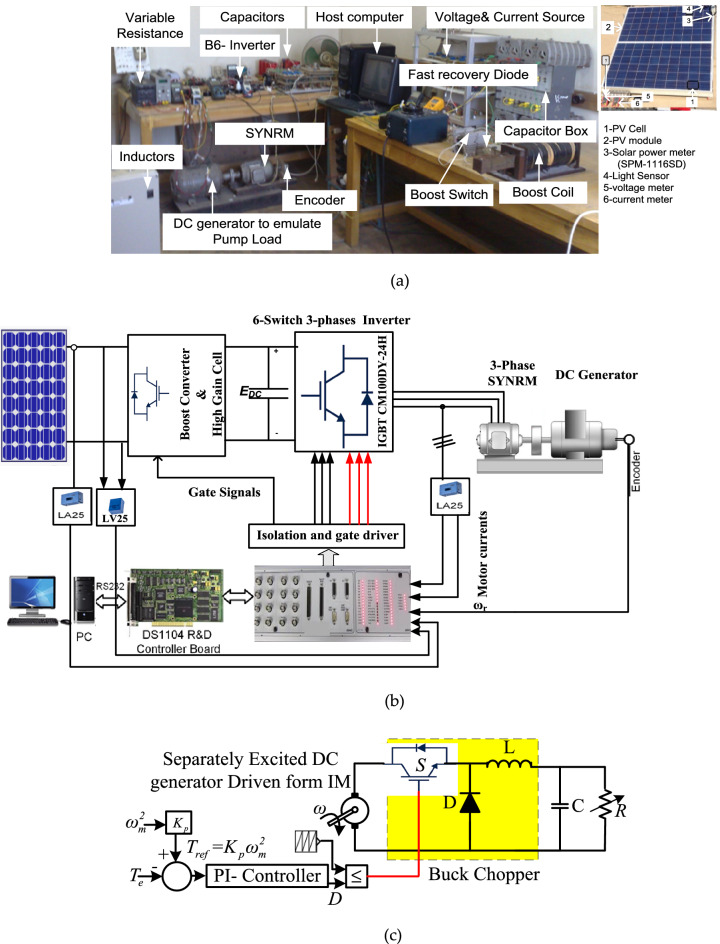
Setup of the laboratory (**a**) wiring diagram, (**b**) implemented image (**c**) load simulation for centrifugal pumps.

The proposed technique is tested to confirm its effectiveness according to Fig. 8 configurations. The structure of the experimental setup is depicted in Fig. 8a. Control and power circuits make up the experimental system. PV, DC to DC boot converter with high gain cell, three-phase inverter, and synchronous reluctance motor combined with centrifugal pump as a load. The DSP-DS1104 control board is used to run the whole system and motor control algorithm. An IGBT (type CM50DY-24H), rapid recovery diodes (type DESI 60), capacitors, and coils make up a DC-to-DC converter with a high gain cell. Voltage sensors (LV25-P) detect PV voltage signals, which are scaled down to 10 V and sent to the dSP. The motor speed is sensed and transmitted to the dSPACE encoder port using an incremental pulse encoder. The experimental system's parameters are in the appendix. In addition, a centrifugal pump load was simulated. In order to evaluate the pump's load torque profile, a buck converter is attached between the DC generator and the resistive load. By comparing the command torque with the electromagnetic torque of the induction motor, 'Te', the control pulses of this buck converter are generated.

Figure 8b shows the procedure of this setup works. The reference torque is calculated by taking the square of the induction motor speed and multiplying it by the motor torque constant, 'K'. A torque controller, which is just a PI controller, is used to decrease the error between these two torques. Finally, the firing pulses for the buck converter are generated by comparing the torque controller output with a specified frequency sawtooth signal, as shown in Fig. 8c.

## Results and discussions

The system is tested using simulation and validated experimentally to ensure the control system. The overall system is simulated using MATLAB/SIMULINK. The converter with high cell components, motor and load parameters of the proposed scheme are listed in the Appendix. The proposed system with the high control system is tested under constant and varied solar irradiation levels to prove the effeteness of the proposed scheme experimentally. Two simulation cases studied are defined as Case 1, where the dynamic system response is tested at constant irradiation of 1000 W/m2 while Case 2, the system dynamic response, is tested under varied irradiation levels. In addition, two experimental-based dynamic system verifications are carried out at 1000 and 800 W/m^2^ for Cases 3 and 4.

### Simulation results for cases 1 and 2

**Figure 9:**
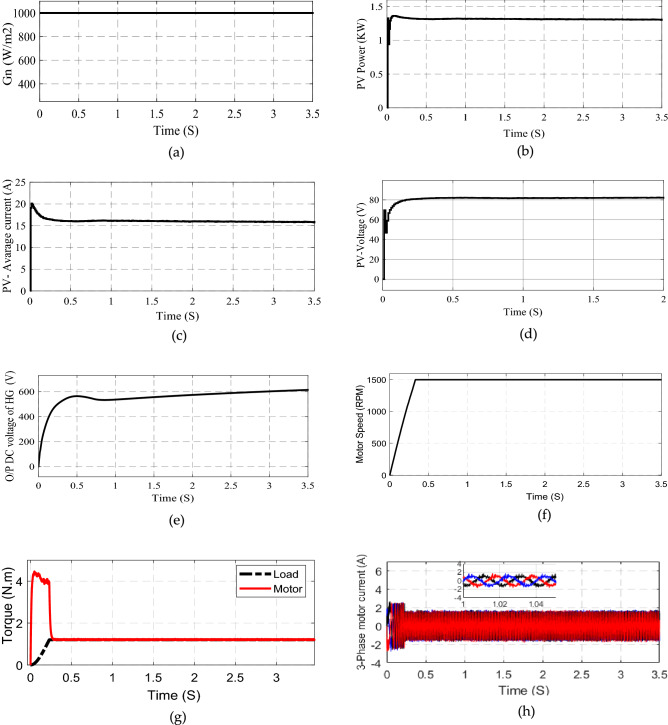
System starting response (**a**) Irradiation (**b**) PV-power (V) PV current (**d**) PV-voltage (**e**) Voltage after high gain cell (**f**) Motor speed (**g**) Motor and load torque (**h**) Motor phase current.

Figure 9. shows the system response at constant solar Irradiation 1000 W/m^2^ in case 1. Figure 9a shows the solar irradiation. Figure 9b–d illustrates the output of solar panel power, voltage, and current is constant. Figure 9e shows the output of the high gain cell. Figure 9f shows the motor speed it reaches to constant value without oscillation. Figure 9g shows the motor and pump torque signals, the motor torque covers the pump torque during the statement period, which introduces a good performance of the drive system algorithm. Figure 9h shows motor phase current with constant amplitude and frequency.

**Figure 10:**
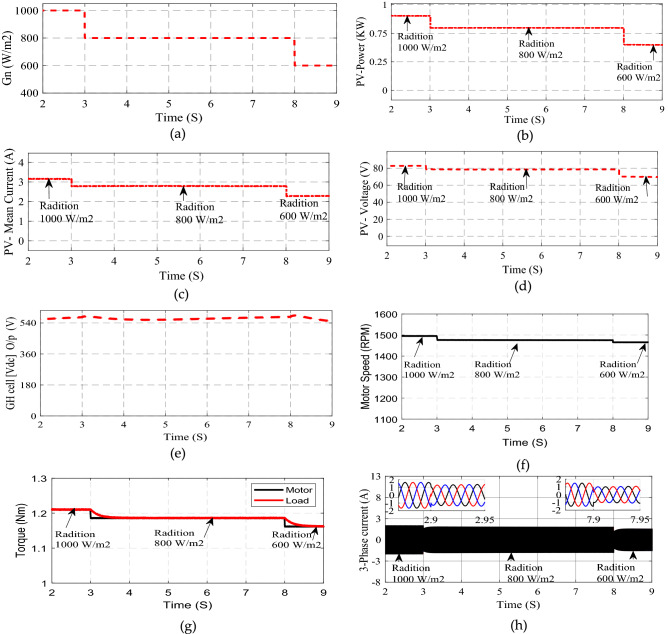
System response at varied Irradiation levels (**a**) Irradiation (**b**) PV-power (V) PV current (**d**) PV-voltage (**e**) Voltage after high gain cell (**f**) Motor speed (**g**) motor and load torque (**h**) Motor phase current.

In case 2, Fig. 10 demonstrates the system response at variation of solar irradiation, decreases from 1000 to 800 W/m^2^ at t = 3 s and it decreases again from 800 to 600 W/m^2^ at t = 8 s Fig. 10a illustrates the solar irradiation. Figure 10b–d shows the output of solar panel power, voltage and current it decreases due to variation of solar irradiation. Figure 10e demonstrates the high gain cell output voltage it stepped up about 7 times of PV panel output voltage. Figure 10f shows the motor speed it decreases with Irradiation decreases and settled to constant value without fluctuation. Figure 10g shows the motor and pump torque signals, the motor torque decreases with irradiation changes due to changes in PV output voltage, also, the pump torque changes due change of motor speed, the changes in motor and load torque illustrate good interconnected of dive system with load. Figure 10h shows motor phase current with constant amplitude and variable frequency results from motor speed variations.

### Experimental verifications

**Figure 11:**
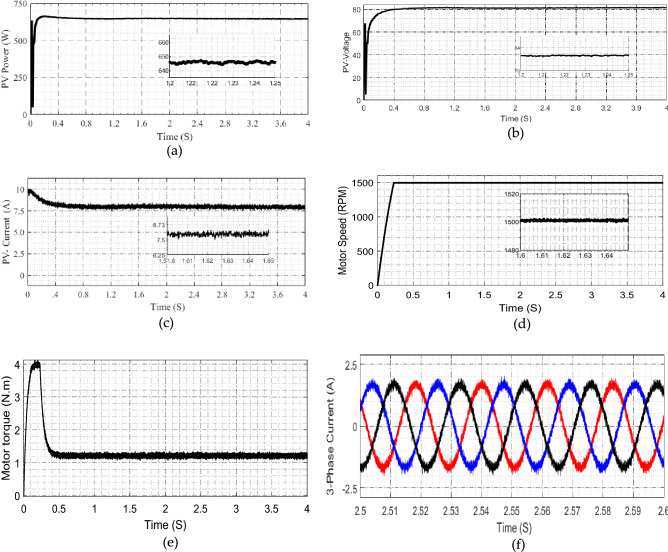
Experimental results at Irradiation 1000 W/m^2^ (**a**) PV-power (**b**) PV-voltage (**c**) PV-current (**d**) voltage after high gain cell motor speed (**e**) motor torque (**f**) motor phase current.

The implemented system is tested experimentally at two conditions of irradiation at 1000 W/m^2^ and at 800 W/m^2^ for Cases 3 and 4. In Case 3, Fig. 11 demonstrates the system response at 1000 W/m^2^ of solar irradiation, Fig. 11a–c show the output of solar panel power, voltage and current, respectively. Figure 11d shows the motor speed, it settled to constant value without variation. Figure 11e illustrates the motor torque where Fig. 11f shows the motor phase currents.

**Figure 12:**
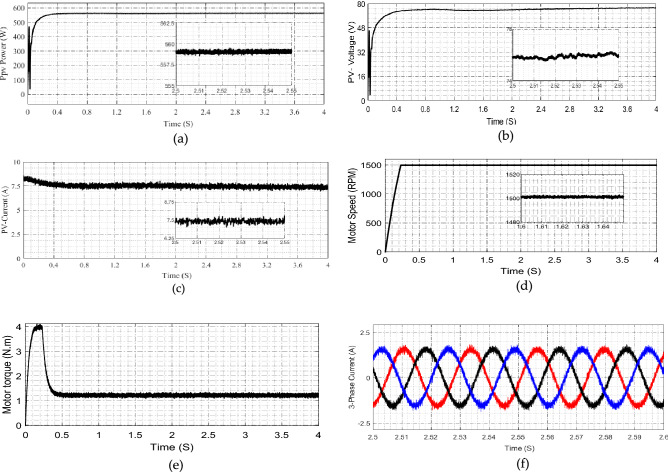
Experimental results at Irradiation 800 W/m^2^ (**a**) PV-power (**b**) PV-voltage (**c**) PV-current (**d**) voltage after high gain cell motor speed (**e**) motor torque (**f**) Motor phase current.

Similarly in Case 4, Fig. 12 shows the experimental results at 800/m^2^ of solar irradiation, Fig. 12a–c illustrates the solar panel production power, voltage and current. Figure 12d shows the motor speed, it fell to persistent value without variation. Figure 12e illustrates the motor torque where Fig. 12f shows the motor phase currents.

## Conclusions

This paper presents photovoltaic fed Synchronous Reluctance motor drive for water pumping system based on a high gain boost converter. Considering the modification in the typical system, the proposed system stepped up the output voltage of photovoltaic system around 7 times. The stepped-up voltage supplied to the DC to three phase voltage source inverter which efficiently drives the asynchronous motor for water pumping system. Additionally, the proposed system is tested at different values of irradiation. The simulation and experimental setup are developed for the high gain cell converter and AC drive scheme and the results validated the effectiveness of proposed scheme. For the experimental setup, the boost converter with high gain cell control and IRFOC has been implemented on the DSP-DS1104 control board. Furthermore, the proposed control scheme is evaluated under various irradiation conditions and the suggested approach has the subsequent benefits: larger voltage increase, decrease ripple, switch stress, cost of converters, and reduces the variations at the photovoltaic operation point.

## Supplementary Information

Supplementary Information.

## Data Availability

Derived data supporting the findings of this study are available from the corresponding author on request.

## References

[1] Lynn PA (2011). Electricity from Sunlight: An Introduction to Photovoltaics.

[2] Meng T (2014). Terawatt Solar Photovoltaics Roadblocks and Opportunities.

[3] Waly, H. M., Osheba, D. S., Azazi, H. Z., & El-Sabbe, A. E. Induction motor drive for PV water-pumping system with high-gain non-isolated DC–DC converter. In *2019 IEEE Conference on Power Electronics and Renewable Energy (CPERE)* 497–503. (IEEE, 2019).‏

[4] Ayodele TR, Ogunjuyigbe ASO, Ekoh EE (2016). Evaluation of numerical algorithms used in extracting the parameters of a single-diode photovoltaic model. Sustain. Energy Technol. Assess..

[5] Parveen, H., Sharma, U. & Singh, B. Battery supported solar water pumping system with adaptive feed- forward current estimation. In *IEEE Transactions on Energy Conversion*. 10.1109/TEC.2022.3147496.

[6] Prabhakaran KK, Karthikeyan A, Varsha S, Perumal BV, Mishra S (2020). Standalone single stage PV-fed reduced switch inverter based PMSM for water pumping application. IEEE Trans. Ind. Appl..

[7] Tripathi PR, Thakura PR, Laxmi V, Keshri RK (2021). Stand-alone PV water pumping system based on high-gain resonant inverter fed induction motor serving two-head for permanent water supply. Int. J. Circ. Theor. Appl..

[8] Nassar-Eddine I, Obbadi A, Errami Y, Agunaou M (2016). Parameter estimation of photovoltaic modules using iterative method and the Lambert W function: A comparative study. Energy Convers. Manag..

[9] Bose, B. K. *Power Electronics and Motor Drives: Advances and Trends*. (Academic Press, 2020).

[10] Salem EZ (2021). High gain boost converter fed asynchronous motor drive for water pumping system. Int. J. Electron..

[11] De Brito MAG, Galotto L, Sampaio LP, e Melo GDA, Canesin CA (2012). Evaluation of the main MPPT techniques for photovoltaic applications. IEEE Trans. Ind. Electron..

[12] Zaky AA, Ibrahim MN, Rezk H, Christopoulos E, El Sehiemy RA, Hristoforou E (2020). Energy efficiency improvement of water pumping system using synchronous reluctance motor fed by perovskite solar cells. Int. J. Energy Res..

[13] Xu L, Yao J (1992). A compensated vector control scheme of a synchronous reluctance motor including saturation and iron losses. IEEE Trans. Ind. Appl..

[14] Park, J., Hofmann, H., & Khalizadeh, C. Feedforward control of high-speed solid-rotor synchronous reluctance machines with rotor dynamics model. In *Proc. 39th IEEE IAS Annu. Meeting* 292–298 (2004).

[15] Saravanan S, Babu NR (2017). Analysis and implementation of high step-up DC–DC converter for PV based grid application. Appl. Energy.

[16] Maalandish M, Hosseini SH, Jalilzadeh T, Vosoughi N (2018). High step-up DC–DC converter using one switch and lower losses for photovoltaic applications. IET Power Electron..

[17] Ardi H, Ajami A, Sabahi M (2017). A novel high step-up DC–DC converter with continuous input current integrating coupled inductor for renewable energy applications. IEEE Trans. Ind. Electron..

[18] Hu X, Gong C (2014). A high gain input-parallel output-series DC/DC converter with dual coupled inductors. IEEE Trans. Power Electron..

[19] Al-Saffar MA, Ismail EH (2015). A high voltage ratio and low stress DC–DC converter with reduced input current ripple for fuel cell source. Renew. Energy.

[20] Lee S, Kim P, Choi S (2012). High step-up soft-switched converters using voltage multiplier cells. IEEE Trans. Power Electron..

[21] Tofoli FL, de Souza Oliveira D, Torrico-Bascopé RP, Alcazar YJA (2012). Novel nonisolated high-voltage gain dc–dc converters based on 3SSC and VMC. IEEE Trans. Power Electron..

[22] Xiong S, Tan SC, Wong SC (2012). Analysis and design of a high-voltage-gain hybrid switched-capacitor buck converter. IEEE Trans. Circuits Syst. I Regul. Pap..

[23] Khadmun W, Subsingha W (2013). High voltage gain interleaved dc boost converter application for photovoltaic generation system. Energy Proc..

[24] Nouri T, Hosseini SH, Babaei E, Ebrahimi J (2014). Generalised transformerless ultra step-up DC–DC converter with reduced voltage stress on semiconductors. IET Power Electron..

[25] Lin CC, Yang LS, Wu GW (2013). Study of a non-isolated bidirectional DC–DC converter. IET Power Electron..

[26] Nouri T (2014). Interleaved high step-up DC–DC converter based on three-winding high-frequency coupled inductor and voltage multiplier cell. IET Power Electron..

[27] Sabzali AJ, Ismail EH, Behbehani HM (2015). High voltage step-up integrated double Boost–Sepic DC–DC converter for fuel-cell and photovoltaic applications. Renew. Energy.

[28] An L, Lu DDC (2014). Design of a single-switch DC/DC converter for a PV-battery-powered pump system with PFM+ PWM control. IEEE Trans. Ind. Electron..

[29] Taheri B, Aghajani G, Sedaghat M, Sharifi S, Teimourian M (2019). Capacitor switching-based high-gain boost chopper converters analysis. Majlesi J. Energy Manag..

[30] Sathishkumar S, Nagarajan R, Yuvaraj R, Sridhar M, Elangovan M (2018). Implementation of PWM technique for integrated high gain boost resonant converter. IOSR J. Eng. IOSRJEN.

[31] Bayoumi AS, El-Sehiemy RA, Mahmoud K, Lehtonen M, Darwish MM (2021). Assessment of an improved three-diode against modified two-diode patterns of MCS solar cells associated with soft parameter estimation paradigms. Appl. Sci..

[32] Said M, Shaheen AM, Ginidi AR, El-Sehiemy RA, Mahmoud K, Lehtonen M, Darwish MM (2021). Estimating parameters of photovoltaic models using accurate turbulent flow of water optimizer. Processes.

[33] Ginidi A, Ghoneim SM, Elsayed A, El-Sehiemy R, Shaheen A, El-Fergany A (2021). Gorilla troops optimizer for electrically based single and double-diode models of solar photovoltaic systems. Sustainability.

[34] Bayoumi ASA, El-Sehiemy RA, Abaza A (2021). Effective PV parameter estimation algorithm based on marine predators optimizer considering normal and low radiation operating conditions. Arab. J. Sci. Eng..

[35] Singh B, Sharma U, Kumar S (2018). Standalone photovoltaic water pumping system using induction motor drive with reduced sensors. IEEE Trans. Ind. Appl..

[36] Bendib B, Belmili H, Krim F (2015). A survey of the most used MPPT methods: Conventional and advanced algorithms applied for photovoltaic systems. Renew. Sustain. Energy Rev..

[37] Sontake VC, Kalamkar VR (2016). Solar photovoltaic water pumping system—A comprehensive review. Renew. Sustain. Energy Rev..

[38] Elgendy MA, Atkinson DJ, Zahawi B (2016). Experimental investigation of the incremental conductance maximum power point tracking algorithm at high perturbation rates. IET Renew. Power Gener..

[39] Shukla S, Singh B (2018). Single-stage PV array fed speed sensorless vector control of induction motor drive for water pumping. IEEE Trans. Ind. Appl..

[40] Yang LS, Liang TJ, Lee HC, Chen JF (2010). Novel high step-up DC–DC converter with coupled-inductor and voltage-doubler circuits. IEEE Trans. Ind. Electron..

