# Supplementary material for: High gain chopper supplied from PV system to fed synchronous reluctance motor drive for pumping water application
Source: Sci Rep. 2022 Sep 15;12:15519. doi: 10.1038/s41598-022-19671-x (PMC9477869; doi:10.1038/s41598-022-19671-x)
Supplement: Supplementary file 1 — Supplementary Information. [file 41598_2022_19671_MOESM1_ESM.docx]

**Appendix**

**Table A1. Tested** system parameters.

| synchronous **reluctance** Motor Parameters | | | | | |
| --- | --- | --- | --- | --- | --- |
| Power | 750 W | d-axis inductance (Ld) | | | 0.4552 H |
| Phase voltage | 220 V | q-axis inductance (Lq) | | | 0.1432 H |
| Frequency | 50 Hz | Friction coefficient: Bm | | | 0.0001 |
| Poles | 4 | Stator resistance (rS)­ | | | 14 Ω |
| Connection | Star | Iron loss Resistance: Rm | | | 1000 Ω |
| inertia (J) | .001 kg.m2 | Motor speed (RPM) | | | 1500 |
| High Gain Parameters | | | | | |
| L1 | 10mH | L2 | 10mH | L3 | 1mH |
| C | 22µF | C1 | 2.2µF | CM | 0.1µF |
| C0 | 220µF |  | | | |
| PV Module and Array Specifications | | | | | |
| Short circuit Current of Module | | | | 𝐈𝐬𝐜 | 4.63A |
| \| M.P.P. Current of Module \| \| --- \| | | | | 𝐈𝐦𝐩 | 4.15A |
| \| Open circuit Voltage of Module \| \| --- \| | | | | 𝐕𝐨𝐜 | 37.4V |
| \| MPP Voltage of Module \| \| --- \| | | | | 𝐕𝐦𝐩 | 30.7V |
| MPP Array Voltage | | | | 𝐕𝐦𝐩 | 105V |
| MPP Array Power | | | | Pmp | 4 kW |
| \| No of Series Modules \| \| --- \| | | | | 𝐍𝐬 | 4 |
| \| No of Parallel Modules \|  \| \| --- \| --- \| | | | | 𝐍𝐩 | 1 |
